# Evidence based QUality Improvement for Prescribing Stewardship in ICU (EQUIPS-ICU): protocol for type III hybrid implementation-effectiveness study

**DOI:** 10.1186/s13012-024-01413-4

**Published:** 2025-02-25

**Authors:** Duncan Wagstaff, John Amuasi, Sumaiya Arfin, Diptesh Aryal, Mohd Basri Mat Nor, Joseph Bonney, Arjen Dondorp, David Dongelmans, Layoni Dullawe, Fathima Fazla, Aniruddha Ghose, Eva Hanciles, Rashan Haniffa, Madiha Hashmi, Adam Hewitt Smith, Bharath Kumar, Yen Lam Minh, Ramani Moonesinghe, Luigi Pisani, Cornelius Sendagire, Mohd Shahnaz Hasan, Maryam Shamal Ghalib, Moses Siaw Frimpong, Otavio Ranzani, Menbeu Sultan, David Thomson, Swagata Tripathy, Louise Thwaites, Rabiul Alam Md. Erfan Uddin, Mohd Zulfakar Mazlan, Wangari Waweru-Siika, Abigail Beane

**Affiliations:** 1 Centre for Preoperative Medicine, University College London, London, UK; 2 Department of Global Health, Kwame Nkrumah University of Science and Technology, Kumasi, Ghana; 3 The George Institute for Global Health, New Delhi, India; 4 Amsterdam Public Health (APH), Amsterdam University Medical Centre(UMC), Amsterdam, The Netherlands; 5 Mahidol Oxford Tropical Medicine Research Unit, Bangkok, Thailand; 6 Department of Critical Care, Nepal Intensive Care Research Foundation, Kathmandu, Nepal; 7 Department of Intensive Care Anaesthesiology, International Islamic University Malaysia, Kuala Lumpur, Malaysia; 8 Emergency Medicine Department, Komfo Anokye Teaching Hospital, Kumasi, Ghana; 9 Amsterdam Institute for Global Health and Development, Amsterdam, The Netherlands; 10 Nuffield Department of Medicine, University of Oxford, Oxford, UK; 11 National Intensive Care Evaluation (NICE) Foundation, Amsterdam, The Netherlands; 12 Department of Intensive Care Medicine, UMC, University of Amsterdam, Amsterdam, The Netherlands; 13 Nat-Intensive Care Surveillance, Mahidol Oxford Tropical Medicine Research Unit, Colombo, Sri Lanka; 14 Department of Medicine, Chittagong Medical College Hospital, Chattogram, Bangladesh; 15 Connaught Hospital, Freetown, Sierra Leone; 16 Ministry of Health and Sanitation, Freetown, Sierra Leone; 17 Pandemic Sciences Hub and Institute for Regeneration and Repair, University of Edinburgh, Edinburgh, UK; 18 Department of Critical Care Medicine, Ziauddin University, Karachi, Pakistan; 19 Faculty of Health Sciences, Busitema University, Mbale, Uganda; 20 William Harvey Research Institute, Queen Mary University of London, London, UK; 21 Department of Critical Care Medicine, Apollo Hospitals Educational and Research Foundation, Chennai, India; 22 Clinical Research Unit, Oxford University, University of Oxford, Ho Chi Minh City, Vietnam; 23 D’Or Institute for Research and Education, Sao Paulo, Brazil; 24 Uganda Heart Institute, University of Makerere, Makerere, Uganda; 25 General Surgery, Wazir Akbar Khan Hospital, Kabul, Afghanistan; 26 Department of Anaesthesiology and Intensive Care, Komfo Anokye Teaching Hospital, Kumasi, Ghana; 27 Barcelona Institute for Global Health, ISGlobal, Barcelona, Spain; 28 Pulmonary Division, Heart Institute, Faculty of Medicine, Hospital das Clínicas da Faculdade de Medicina da Universidade de São Paulo, São Paulo, Brazil; 29 Department of Emergency Medicine and Critical Care, St. Paul’s Hospital Millennium Medical College, Addis Ababa, Ethiopia; 30 Department of Anaesthesia and Perioperative Medicine, University of Cape Town, Cape Town, South Africa; 31 AII India Institute of Medical Sciences, New Delhi, India; 32 Hospital Universiti Sains Malaysia, Kota Bharu, Malaysia; 33 Department of Anaesthesia, The Aga Khan University, Nairobi, Kenya; 34 Department of Implementation Research, Bernhard Nocht Institute of Tropical Medicine, Hamburg, Germany; 35 Global Health and Infectious Disease Research Group, KCCR, KNUST, Kumasi, Ghana

**Keywords:** Antimicrobial Stewardship, Antimicrobial Resistance, Implementation, Intensive Care, Critical Care, Low- and Middle- Income Countries, Quality Improvement, Audit & Feedback

## Abstract

**Background:**

Approximately half of all antimicrobial prescriptions in intensive care units (ICUs) may be inappropriate, including those prescribed when not needed, in unnecessary combinations or for longer durations than needed. Inappropriate prescribing is costly, exposes patients to unnecessary side-effects and drives population-level antimicrobial resistance, the prevalence and consequences of which are greatest in low- and middle-income countries. However, the implementation of interventions to improve the appropriateness of antimicrobial prescribing has been variable and requires further study.

**Methods:**

We propose a type III hybrid implementation/effectiveness interventional cohort trial in 35 ICUs in up to 11 low- and middle- income countries. The study intervention is a structured review of antimicrobial prescriptions as recommended by the World Health Organisation. Strategies to support stakeholder-led implementation include development of local protocols, registry-enabled audit and feedback, and education. Evaluation of implementation, and the determinants of its success, is informed by the RE-AIM framework and the Consolidated Framework for Implementation Research respectively. The primary outcome is a composite measure of fidelity, reach and adoption. Secondary outcomes describe the effectiveness of the intervention on improving antimicrobial prescribing. Qualitative interviews will assess relevant implementation acceptability, adaptations and maintenance. A baseline survey will investigate ICU-level antimicrobial stewardship structures and processes.

**Discussion:**

This study addresses global policy priorities by supporting implementation research of antimicrobial stewardship, and strengthening associated healthcare professional competencies. It does this in a setting where improvement is sorely needed: low- and middle- income country ICUs. The study will also describe the influence of pre-existing antimicrobial stewardship structures and processes on implementation and improve understanding about the efficacy of strategies to overcome barriers to implementation in these settings.

**Trial registration:**

This study protocol has been registered with ClinicalTrials.gov (ref NCT06666738) on 31 Oct 2004. https://clinicaltrials.gov/study/NCT06666738?term=NCT06666738&rank=1.

**Supplementary Information:**

The online version contains supplementary material available at 10.1186/s13012-024-01413-4.

Contributions to the literature

- This protocol aims to implement structured antimicrobial reviews in ICUs across XX LMICs
- An evidence-cased multifaceted implementation strategy is used involving local protocols, audit and feedback, and online education
- This protocol uses mixed-methods to evaluates the primary outcome of implementation alongside secondary outcomes of intervention effectiveness
- Findings have the potential to improve local implementation capacity, inform implementation science in similar settings, and advance progress in a priority clinical topic of antimicrobial stewardship.

## Background

Antimicrobial Stewardship (AMS) describes a diverse set of programmes and projects aimed at reducing inappropriate antimicrobial use [1]. Improving AMS is a global health priority in order to maximise treatment and prevention of infections, minimise the spread of Antimicrobial Resistance (AMR), and minimise adverse drug events [2]. In 2019 it was estimated 4.95 million deaths globally were associated with AMR [3], and this is predicted to rise to 10 million deaths annually with a cumulative $100 trillion loss of economic output by 2050 [4].

The intensive care unit (ICU) is an important in-hospital setting in which to optimise AMS since an estimated 70% of ICU patients receive antimicrobials during the intensive care admission. An estimated 50% of ICU patients have diagnosed bacterial infections (of which many are hospital-acquired infections), many patients are admitted to the ICU having already received often more than one antimicrobial agent, and many others have septic shock where rapid administration of broad-spectrum antibiotics is both indicated and common [1, 5, 6]. As a consequence of the acute illness of ICU patients, concomitant ICU therapies which suppress immunity and the high healthcare-worker to patient ratio risking cross infection, there is a high density of multi-drug-resistant organisms in ICU [7]. These population, treatment process and environmental factors makes delivering appropriate antimicrobial therapy in ICU settings challenging. Approximately 40–50% of ICU antimicrobial prescribing is ‘inappropriate’ [5, 8, 9], and is associated with increased ICU mortality [10]. The most common examples of antimicrobial inappropriateness include: overprescribing (antimicrobials prescribed which are not needed); unnecessary combination therapy; wrong antibiotic choice for documented indication; incorrect route of administration (e.g. parenteral instead of enteral); and duration of therapy longer than necessary [11, 12].

The prevalence and consequences (both direct and indirect) of AMR are greatest in Low- and Middle- Income Countries (LMICs) [4]. Hospital acquired infections, especially with antimicrobial resistant organisms, are more common in LMIC ICUs, making the need for AMS even higher [13]. These infections are a major drive of healthcare expenditure, both for providers and patients [14]. Drivers of inappropriate antimicrobial use in LMICs, many of which are also present in High Income Countries [15], include: more common use of open (or semi-closed) ICU designs [16]; over the counter availability of antimicrobials; public expectations regarding escalation and duration of treatment; lack of diagnostic capacity [17]; unrestricted and/or inconsistent access to antimicrobials; shortages in staffing/resources; reluctance to change prescribing behaviours; and exclusion of multidisciplinary team members from interventions and/or decision-making [18]. These drivers manifest in clinical practice as increased use of broad spectrum antimicrobials, duplication of agents for the same indication, continuation of therapies in the absence of confirmed infection, and prolonged treatment durations or reluctance to cease therapy [19, 20]. Whilst these challenges are not unique to LMICs, their prevalence combined with poorer access to laboratory and microbiology services, compounds the problem [21].

There is strong evidence of the impact of AMS interventions in in-hospital settings. A Cochrane review found high-certainty evidence that AMS increased compliance with local prescribing policies, reduced durations of antimicrobials, and probably reduced lengths of stay without increasing mortality [22]. However, the success and impacts of implementing AMS internationally have been variable [23, 24]. One of several challenges in synthesising and interpreting the findings from the existing literature of AMS interventions in hospital settings is the multiplicity of indicators (mainly structural and process) that have been used to evaluate both implementation effectiveness and practice change [25]. The Cochrane review concluded that further AMS research should aim to include assessment of different stewardship interventions and their implementation [22].

The World Health Organisation (WHO) recommendations for AMS in LMICs seek to address inappropriate prescribing, promote multidisciplinary engagement, and establish systems for audit and feedback [11]. Whilst interventions aiming to improve stewardship in LMICs are widely reported, their success to date has been variable. In the ICU setting, both implementation and intervention effectiveness has been limited. Foreseen and unforeseen gaps between intervention design and the existing ICU prescribing practices include missing local information on antimicrobial resistance, disconnects between physicians and other medical staff, patient pressure to prescribe antimicrobials, lack of electronic health records, a lack of local resources or governance structures to support stewardship [17]. These gaps, combined with differences in organisational structures, care processes and individual behaviours, act as direct barriers to intervention adoption and practice change [22, 26].

## Methods

### Project objectives and hypotheses

This project seeks to determine whether a QI intervention to improve appropriateness of antimicrobial prescribing can be implemented in LMIC ICUs as part of an existing multinational Care Quality Registry (CQR) network. We hypothesise that a stakeholder co-designed intervention to improve appropriateness of antimicrobial prescribing can be implemented according to a priori thresholds of fidelity, reach and adoption in participating ICUs. The objectives of this project are therefore:

1. To determine whether a structured antimicrobial review can be implemented in LMIC ICUs
2. To evaluate the impact of a structured antimicrobial review on rates of antimicrobial density, redundancy and associated indicators of antimicrobial utilisation.

### Project Design

We report this protocol using the SPIRIT guidelines for reporting intervention trials (Supplementary file 1) [27]. This project is a hybrid implementation—effectiveness design, with the primary outcome being implementation (assessed by fidelity, reach and adoption), and the secondary outcomes being the success of the intervention on improving antimicrobial prescribing (care processes and outcomes). Such hybrid study designs are increasingly advocated in healthcare improvement [28]–[30]. This project uses a person-centred approach [31] whereby national CQR and ICU clinical stakeholders will have ownership of the project within their clinical settings. The implementers are appointed ICU Champions, and they, together with the stakeholders, will be responsible for engagement of sites, navigating institutional administration, providing input into the design and implementation of the intervention, and its subsequent adaptations to achieve adoption into daily practice [31]. The RE-AIM (Reach Effectiveness Adoption Implementation Maintenance) framework will be used to structure the evaluation of the project [32]. This framework provides a widely accepted and reproducible guideline for evaluating implementation studies. Designed to assess both implementation effectiveness and its determinants, RE-AIM also informs future scalability and sustainability. Primary and secondary outcomes will be collected using both quantitative data (via the existing CQR dataset and a project E-CRF) and qualitative data captured through interviews with the ICU Champions.

### Ethical considerations

The project will be conducted in accordance with relevant national and international guidance and regulations, including the Global Code of Conduct for Research in Resource-Poor Settings [56]. To ensure that the project is conducted in an ethical manner, this protocol has been approved by the Oxford Tropical Research Ethics Committee (OxTREC – ref 559–24, Supplementary File 7 [57]). CQR national leads will be responsible for coordinating with their participating sites for any institutional or institute review boards for relevant approvals. Individual patient consent will not be sought as the intervention intends ICU level service improvement in line with international recommendations. All patient level data will be anonymised. Champions will give verbal consent prior to being interviewed and can withdraw their consent at any stage (see Supplementary File 4 – Participant Information Sheet).

### Setting

Collaboration for Research Implementation, Training in Critical Care, Asia Africa (CCAA) is an international network of Care Quality Registries (CQR), spanning 15 countries and 300 + acute and critical care units. Following stakeholder selection of QI indicators in 2022, eleven collaborating registries elected to implement AMS surveillance in their respective CQRs; Afghanistan, Ethiopia, Ghana, India, Kenya, Malaysia, Nepal, Pakistan, South Africa, Uganda and Vietnam.

### Site selection criteria

All adult ICUs within CCAA-affiliated CQRs, where surveillance of antimicrobial utilisation has already been established will be considered for participation. ICUs must appoint a named Champion in order to participate. A survey (Supplementary File 2) will be conducted to determine pre-existing AMS processes and current adoption of structured review activities. Pre-existing antimicrobial review processes are neither a requirement for an ICU to participate nor an exclusion criterion. Instead, parallel to the survey, pre-implementation patient-level data pertaining to current antimicrobial prescribing review practices will be measured during months 1–2. ICU’s which are found to already have a structured antimicrobial review in place, and which demonstrate adoption and reach of 80% or greater (median over the 2 months) will not be eligible to participate in the implementation. This is because the baseline activity will confound the selected measures for implementation success. However, findings of the project will be shared with those ICUs, along with opportunities to participate in subsequent improvement activities.

### Recruitment

We anticipate recruiting between 35–40 ICUs. Using existing CQR data from collaborating registries, we estimate that 75% of adult patients admitted to ICUs receive one or more antimicrobials. ICU occupancy varies between ICUs, but we estimate a median admission rate of 40 patients per month. Therefore, we expect a median inclusion of 180 ICU patient encounters for each ICU during the 6-month period, (totalling approximately 6300 ICU encounters).

Interviews with ICU Champions are described below. One interview with each Champion will be conducted during months 4–6, and one following month 6, resulting in 70 interviews (35 ICUs*2) and 3 months of field notes (recorded during months 4–6). Implementation challenges and effectiveness are likely to vary across sites, so we aim to include qualitative data from all sites. Qualitative data may, however, have a lower sample size as we will stop during each round when we reach saturation or predictability (data will be analysed contemporaneously).

### Intervention: structured review of antimicrobial prescribing appropriateness

#### Rationale

Structured multidisciplinary antimicrobial prescription reviews have been demonstrated to reduce overall antimicrobial utilisation and prescription redundancy (‘two or more agents intentionally, or unintentionally duplicating treatment’) [33, 34]. Redundancy directly impacts antimicrobial resistance rates and escalates healthcare costs for patients, providers and payers [22, 35]. Structured prescribing reviews promote: focused antimicrobial agent choice in response to microbiological evidence; avoidance and/or de-escalation of antimicrobial prescriptions where patients are found to have non-infectious syndromes or colonisation; utilisation of enteral routes of administration where appropriate, and avoidance of duplication of prescriptions [36]. Such interventions have appeal in the ICU setting where, in addition to patients with confirmed infection, many patients also present with acute inflammatory syndromes following surgery, injury, or in exacerbations of chronic disease, resulting in rapid commencement of antimicrobials. These prescriptions often occur prior to ICU admission, and once started, are difficult to de-escalate [5]. The WHO toolkit for AMS in LMICs prioritises reducing unnecessary and redundant antimicrobial prescribing and consists of four key steps for review: choosing therapies to best suit indications; optimising routes of administration; limiting therapy duration; and documenting a planned stop date [11].

#### Conduct of review

Participating ICU teams will be required to undertake a structured review of each antimicrobial prescription for all patients receiving antimicrobials. Reviewing prescriptions allows their appropriateness to be reconsidered in the context of emerging clinical and/or microbiological data [37]. The review should be completed within 48 h of a new prescription being commenced, or at ICU admission where pre-ICU antimicrobial prescriptions exist. The review will necessitate documentation of the four key steps as defined by the WHO: indication (in relation to pre-existing local guidelines), route (e.g. parenteral or enteral), expected duration of therapy and intended stop date (including escalation or de-escalation discussions). It will also necessitate documentation of any changes (or lack thereof) made to the prescription, including: the choice of drug, route of administration and duration of treatment. The reviewing ICU team will consider the choice of therapy in relation to the known or assumed indication, the dose in relation to local ICU antimicrobial guidelines, the route of administration, and whether escalation to parenteral, or de-escalation to enteral route (and related dosing and duration adjustments) are warranted. Given the rapidly changing clinical status of critically ill patients, and emerging clinical and/or microbiological data during the ICU stay, it is anticipated that patients may receive more than one review during their ICU encounter. ICUs’ existing antimicrobial guidelines for the management of infections in critically ill patients will be utilised.

Who leads the review, which multidisciplinary members participate, when it occurs during the ICU working day and how frequently it occurs during a patient’s admission will be for the ICU team to decide during the pre-implementation period. The ICU team, together with the Champion will be encouraged to align the intervention with their existing ICU structures (ward rounds, microbiology rounds, etc.), to maximise feasibility and minimise disruption to existing workflows. Given that patient management in the ICU is most commonly led by the consulting clinicians and decision-making often occurs during daily ward rounds, we anticipate that these existing structures will be used, so as to align with existing clinical roles and responsibilities, and to minimise additional workload. Champions will encourage ICU teams to include pharmacists and microbiologists in the review if these specialists are available as the attendance of these team members is associated with improved prescribing practices [38]. How and where the review decisions are documented, will be decided by the ICU team and where possible use existing documentation practices already operational in the ICU. The information must however be accessible to the ICU Champion and Research Assistant (RA) for daily review during the project.

### Implementation

#### Design

A multifaceted approach has been shown to be a critical condition for success of implementation in various settings [39]. Combining implementation strategies has been demonstrated to have a greater success of both implementation and intervention effectiveness when compared to the use of single strategies alone [40]. This project will combine three implementation strategies that have been reported to have been both effective in implementing practice change in critical care in LMICs in a recent systematic review [39], and leverage existing infrastructure already operational in the CQR networks [41, 42]. These strategies are described below and summarised in Table 1.

**Table 1:** Implementation strategies

QI strategy	Description
1 Protocolised review	Agreement and documentation of a local protocol for structured antimicrobial review (‘the intervention’) in the ICU
2 CQR enabled audit and feedback	CQR report of audit data on implementation of the prescribing review and on prescribing indicators will be available to each ICU. The A&F implementation strategy will augment the existing audit and feedback mechanism which is part of CQR participation. **The existing monthly CQR report has data on:** Proportion of patients receiving one or more antimicrobials (APR), Duration of therapy (DOT) Case mix, care processes and clinical outcomes (Length of Stay, Mechanical Ventilation, mortality). **Additional data collected for audit throughout the project:** Proportion of prescriptions with a documented indication Antimicrobial Density (AD) Culture rates Antimicrobial resistance rates Antimicrobial redundancy rates (as a proportion of total prescriptions). **Additional data collected for audit and feedback weekly during months 4–6:** Proportion of prescriptions reviewed Proportion of reviews within 48 h of prescription commencement Proportion of prescriptions with a documented indication Antimicrobial Density (AD) Proportion of patients with culturing performed Antimicrobial resistance rates (as a proportion of cultures performed). **Ongoing additions to monthly CQR reports beyond month 6** Proportion of prescriptions reviewed
3 Educational resource	Online educational material to support implementation. It will describe: • Protocol of antimicrobial review (who; when; where documented; link to local prescribing guideline) • AMS reports (definition and rationale for metrics; data collection; analysis; interpretation; suggested actions) • Quality improvement techniques

##### Strategy 1

is agreement and documentation of a local protocol for structured review of antimicrobial prescriptions, as described above (’Conduct of review’). The protocol must include the core aspects which the WHO recommends for review of prescribed antimicrobials [11, 43], but will also include adaptations to the local contexts. Expected adaptations include: which team members perform the review; when during the ICU day the review is performed; how frequently the review is undertaken (beyond the mandate of 48 h post prescription); how the review and its conclusions will be documented and disseminated; when the review should be repeated or revisited.

##### Strategy 2

is a registry-enabled audit and feedback cycle. Audits of clinical practice with feedback to clinicians have been shown to improve health outcomes and clinical performance [44]. The WHO has recommended audits to improve antimicrobial prescribing where they are feasible and costs of collecting data are low [11]. By using the existing CQR data and infrastructure we can: 1) provide near-real time feedback on clinical practice and data quality; 2) empower health professionals to act on the feedback and improve clinical practice and data quality; 3) enable efficient health service planning and future research; and 4) automate a large proportion of the data collection for assessing outcomes [45].

Audit and feedback for the project will augment an existing CQR report whereby data on case mix, clinical outcomes, and proportion of patients receiving antimicrobials, along with the following measures of antimicrobial utilisation are reported monthly: Antimicrobial Density (AD), culture availability, Duration of Therapy (DoT) and Antimicrobial Resistance Index (ARI). More details on this dataset are published [42, 45, 46]. For the duration of the project (7 months), existing antimicrobial utilisation reporting will be augmented by the measurement and reporting of antimicrobial redundancy rate. Once structured prescribing review commences, the proportion of eligible patients receiving a prescribing review within 48 h will also be reported, along with each ICU’s performance on the 4 components of the review (indication appropriateness, route, duration and stop date) compared to other participating sites. Data on indications and microbiology data will also be captured and reported. Following commencement of the review, frequency of reports will be increased to weekly (for a maximum of 3 months). Champions will be encouraged to facilitate review of the report and discussion of the audit data with the wider ICU team, at their preferred frequency and structure, but as a minimum weekly. The presence, format and structure of the weekly discussions will be reported by the Champions. Feedback from the ICU teams on the report structure will be sought prior to and during implementation.

##### Strategy 3

is an online education resource to support Champions and ICU teams during implementation. Education has been recommended as a ‘persuasive’ AMS intervention [11] and is synergistic when used in conjunction with Audit and Feedback [9, 17, 47]. The resource will be available through an already existing online CCAA platform co-designed with stakeholders in 2020. The platform has a series of QI modules, aimed at supporting clinical teams who are seeking to undertake quality improvement initiatives in their ICU. The material includes interactive teaching methods, self-assessments and case studies from QI interventions implemented already in acute and critical care in LMICs. This material has already been used successfully in published outputs from the CQR network [48]–[50]. For this project, an additional module specifically focused on AMS will be included. The module will explain the rationale underpinning the prescription review process and the WHO AMS LMIC toolkit, including the dimensions of and justification for the prescribing review. The resource will be available to all ICU teams, and the Champions throughout the project period. In month 1, Champions will define a list of eligible staff members who they feel should engage with the education tool in order to support implementation. Engagement will be tracked by measuring the proportion of these staff who access the tool.

### Implementation team members

Each participating ICU will be asked to identify a Champion for the project. This person will have clinical knowledge and will be from allied specialties who are involved in antimicrobial prescribing in the ICU (e.g. pharmacist, doctor, ICU nurse). The Champion will undergo online training in QI strategies, the intervention and its implementation alongside regular mentorship from the project team throughout the project period. The Champion will be responsible for all aspects of coordination with the ICU team, be the ‘implementer’ within the ICU, and the direct communicator with the project team. At the beginning of the project, they will liaise with the ICU team to complete an online survey (Supplementary File 2) to identify the nature of any existing antimicrobial review processes and other core elements of antimicrobial stewardship. They will have responsibility for the organisation of the ICU team, onboarding to the project, and implementation of the reviews. They will facilitate the weekly audit and feedback cycles, and provide support and feedback to the project team identifying, recording, and solving barriers to implementation [17]. They will be supported by a research assistant (RA) at each ICU who will be recruited for a maximum of 8 months, to support the intervention-specific data collection. The RA will work in partnership with the Champion and the existing CQR data collector. The Champion will also be invited to join online sessions to review and reflect on accruing process and outcome data with the project team and Champions from other sites.

### Implementation procedure

**Fig. 1:**
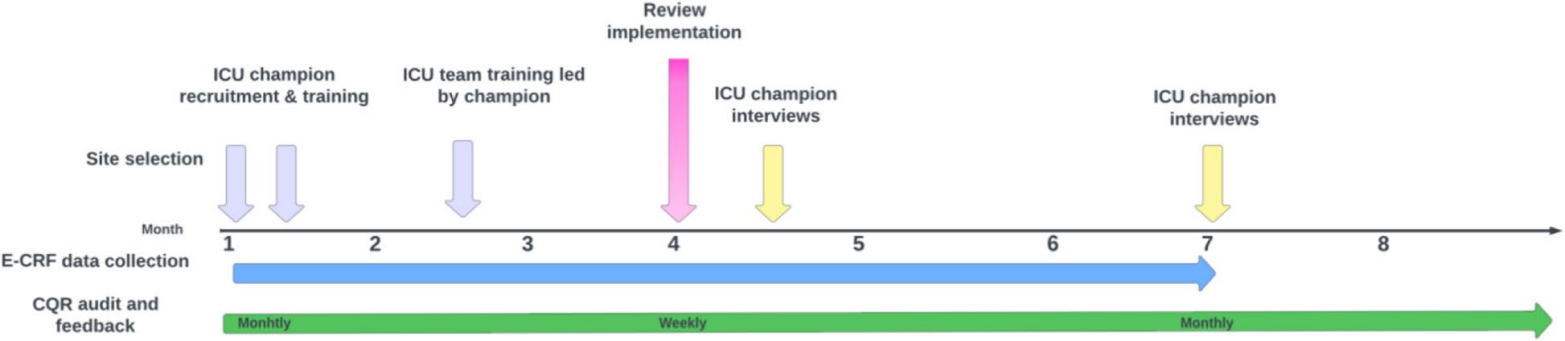
Project timeline

The project timelines are illustrated in Fig. 1. Baseline data collection will occur during months 1–3, during which data pertaining to current antimicrobial utilisation and prescribing appropriateness will be captured daily through the CQR, E-CRF and site survey. During this pre-implementation period, the Champions and RAs will undertake online education and receive training from the project team in review processes and orientation to the data in the A&F reports. In parallel, the Champions will engage the wider ICU team to complete the actions described above. Data on existing prescribing practices, which is already captured as part of the CQR report (see Table 1 below) will remain available to the ICUs.

At month 4, ICU teams will commence the protocolised review in their ICU. The Champion will have access to the online enhanced CQR reports, and the online education material will remain available to all ICU team members. Online support for troubleshooting relating to the implementation strategies will be available to the Champions. In addition, voluntary monthly group sessions will be facilitated by the project team from months 4–6, whereby Champions, RAs and other team members will be welcome to join to reflect on their experience and share learning between sites. Daily data collection (CQR and E-CRF) will continue throughout this period. Champions will be interviewed during months 4–6 to explore barriers and facilitators to implementation, adaptations made to both the intervention and its implementation, along with perceptions regarding likely adoption and maintenance (Supplementary File 3).

At the end of month 6, the implementation strategies of online education, the role of Champions, and the RA collecting daily data on review processes will cease. The daily auditing of whether reviews take place will be transferred to the CQR data collectors and reported monthly as part of the CQR report. A second interview will be conducted with Champions following month 6 to explore barriers and facilitators to adoption, and adaptations to the intervention made post-implementation (Supplementary File 4).

### Adaptations to implementation plan

It is expected that ICU teams will make adaptations to their prescribing review processes. These adaptations will be captured throughout the project by Champions (in implementation logs) and synthesised by the project team as part of the evaluation. Adaptations may also be made to the implementation strategies, with the proviso that any adaptations are made at a ‘project level’, meaning uniformity of materials and resources across all sites. Site or regional level adaptations to implementation strategies will not be made. For example, the structure and/or content of weekly reports may be adapted, or elements may be added to or removed from the online education resource, following feedback. Such adaptations, and the reasons for them, will be logged, explored during interviews and synthesised.

### Outcomes

#### Primary outcome: implementation

Implementation is the primary outcome and will be assessed quantitatively using 3 of the 5 key domains identified through the RE-AIM framework: Reach, Implementation (defined as fidelity) and Adoption (see Table 1). These will be combined into a composite measure of implementation with a threshold of 80% for success in each domain. Furthermore, Champions’ perceptions of the determinants of successful implementation, including acceptability, maintenance of, and adaptations to, implementation will be captured through field notes and interviews [51].

#### Secondary outcomes: Intervention effectiveness

Intervention effectiveness, defined as the intervention's effect on the relevant care processes and clinical outcomes, will be assessed by reporting ICU trends in *prescription appropriateness* (Antimicrobial density (AD), treatment duration (DOT) and antimicrobial redundancy rate (ARR, % of total prescriptions)), and *processes of care* (proportion of patients for whom cultures are sent, antimicrobial resistance rate, antimicrobial prescribing rate and compliance of prescriptions with local guidelines). Clinical care processes and outcomes (mortality, Length of Stay and duration of organ support) will be also collected as safety endpoints. Unintended and/or adverse consequences of the intervention will also be explored through field notes and interviews with Champions.

#### Feasibility of collecting outcomes

Eligible ICUs will have had a CQR established for at least 6 months and have an established CQR data collector. Clinical teams will already be trained in use of the CQR, and in the review and interpretation of CQR reports which will be used for audit and feedback. ICU team members, including the Champion will work together with the existing CQR and clinical teams to ensure that data on implementation and care processes can be integrated into analysis of outcomes. This will include records that include (1) diagnoses, (2) prescriptions of medications, (3) investigations and observations, and (4) care processes associated with AMS and the preselected outcomes. Information on the 4 steps of review (indication, route, expected duration, and expected stop date) will be recorded, either on paper, or electronically and reported as part of the audit and feedback as described above. CQR reports will be generated centrally by the CCAA data coordinating centre and made available to sites through existing mechanisms. Data pertaining to the availability of the above review and CQR information will be used as part of the implementation evaluation.

### Data collection

Data regarding case mix (including source and reason for admission, severity of illness at admission, comorbidities) along with physiology, organ support, antimicrobial prescriptions and microbiology data, care processes (duration of antimicrobials) and clinical outcomes will continue to be collected daily through the existing CQR during months 1–8. The CQR data collectors will also collect adoption and reach of any pre-existing structured review during months 1–2.

A project specific E-CRF on REDcap [52, 53], will capture data on antimicrobial reviews during months 4–6 (see Table 1 above). This additional E-CRF collection will be done by the RAs. Copies of existing antimicrobial guidelines will be requested prior to commencement of the study, and ICU stakeholders’ perspectives regarding the current guidelines, along with information on existing antimicrobial stewardship activities already present in the ICU captured during the site onboarding by Champions. Changes in guidelines made during the project will also be captured, and the current working version of the guidelines queried at each phase of the project. Post implementation (months 7 onwards), the CQR data collection will continue, along with a measure of adoption.

Qualitative data exploring the Champions’ perspectives regarding implementation, acceptability, adoption and maintenance, along with contextual factors influencing implementation will be collected through semi-structured interviews at two time points during the study: implementation (months 4–6) and post-implementation (month > 6). We intend to interview all Champions during both phases but recruitment may cease if concurrent analysis indicates that theoretical saturation has been achieved. Interviews will be conducted remotely using the Zoom (Zoom Video Communications Inc, San Jose, CA, USA) conferencing application. A participant information sheet (Supplementary File 5) will be provided and verbal consent for participation sought (Supplementary File 6). In addition, Champions together with RAs will be requested to maintain an implementation log [54], whereby adaptations, and observed barriers and facilitators to implementation will also be reported. These will be reviewed and thematically coded alongside the interview data.

No site or follow-up visits by the project team are required, as the design of the project is such that ICU teams together with Champions have direct ownership at site level, and the project seeks to minimise additional burden of data collection for clinical staff. All patient level de-identified data will be submitted by collaborating CQRs using existing processes. All data will be inspected before analysis to rule out spurious values, and appropriate transformations implemented where necessary. Existing data quality measures already operational within the CQRs will be utilised to provide information on data availability and reduce erroneous or missed data. These measures are already described in detail and published [55]. Once cleaned, data will be aggregated and anonymised at ICU level for monthly and weekly reporting (audit and feedback) and for the subsequent project analysi.

### Analysis

All analysis will be conducted at the level of ICUs. ICUs will be identifiable only by their type (i.e. medical, surgical, mixed or speciality), and by their organisation governance type (private, public, other). ICUs who have a pre-existing structured review with an adoption rate of 80% will be included in the context description, but not in the intervention analysis described below. No country or regional level analysis is planned. However participating CQRs will have access to their sites’ data for subsequent evaluation once the primary outcome is published.

#### Primary outcome

Successful implementation will be assessed by a composite measure combining rates of fidelity, reach, and adoption. Recent literature describes that for a multifaceted intervention to affect clinical practice change, implementation threshold needs to be 80% [58]–[63]. We will therefore consider this as the threshold for successful implementation at ICU level.

#### Secondary outcomes

For the secondary outcomes of intervention effectiveness, we propose a 10% absolute reduction in Antimicrobial Redundancy Rates (ARR) (Supplementary File 8), Antimicrobial Density (AD), and Duration of Treatment (DoT) (independently), compared with the baseline period, for successful intervention at the ICU level.

#### Statistics

For the primary outcome, we will describe the proportion of ICUs that achieved ≥ 80% of all three indicators (fidelity, reach and adoption). We will also report these indicators separately. We will compare the proportion achieved in the post-implementation period, as specified in the Table 2, with the baseline assessment, using a McNemar test (Chi-square for repeated measures).

**Table 2:** Outcomes

**Outcome**	**Measure and reporting**
Implementation (Primary Outcome)	**Fidelity: **The proportion of prescriptions reviewed as intended (review at 48hrs with documentation of all 4 review components: indication, route, duration and stop date). Reported as a proportion of the number of possible reviews during months 4-6. Numerator: Number of reviews conducted as intended Denominator: Number of prescriptions of antimicrobials Expressed as a %. Binary outcome: success/fail, 80% is threshold. Proportion of weekly A&F report meetings held from month 4 until the time of adoption (Number of meetings/12, expressed as a %) (not in composite outcome)
	**Reach: **Proportion of eligible patients who received a review during months 4-6 Numerator: Number of patients that received at least 1 prescription review Denominator: Number of eligible patients Expressed as a % Binary outcome: success/fail, 80% is threshold. Proportion of eligible staff that receive education (expressed as a %; eligible staff defined prospectively by Champion) (not in composite outcome)
	**Adoption**: Proportion of prescriptions reviewed as intended during month 7 Numerator: Number of prescriptions of antimicrobials reviewed Denominator: Number of prescriptions of antimicrobials Expressed as a % Binary outcome: success/fail, 80% is threshold.
Intervention effectiveness (secondary outcomes)	**Appropriateness of prescriptions:** Antimicrobial Density (AD) Duration of Therapy (DOT) Antimicrobial Redundancy Rate (ARR)
	**Antimicrobial care processes:** Does the drug Duration exceed the Stop date? Antimicrobial resistance rate (% of patients that have an multidrug resistant pathogen identified) Antimicrobial prescribing rate (% of all patients) Count of cultures taken and reported as a proportion of the population on antimicrobials.

For the secondary outcomes, we will perform an interrupted times series analysis. We will observe the weekly temporal resolution of each indicator and set the model to account for the natural/secular time trend, implementation of the intervention (level change) and the trend after implementation (slope change). We will account for auto-correlations. The model applied will depend on the data, but we will evaluate ARIMA models, segmented regressions and Holt-Winters additive model to evaluate the best model for the times series. Counts of actions recorded in response to the review will be described. The code lists for the primary and the secondary outcomes, and definitions for indicators of quality will be developed and published in advance of data collection.

#### Qualitative analysis

Qualitative data will be analysed thematically and contemporaneously to data collection. Analysis of barriers and facilitators to implementation will be informed by the Consolidated Framework for Implementation Research (CFIR) framework [64]. Transcripts of the interviews will be deductively analysed independently by two researchers (DW and AB) to identify the key concepts (themes) regarding acceptability and maintenance of implementation, along with barriers and facilitators to implementation. DW and AB have extensive experience of working in ICU clinically and as researchers internationally. Initially the text will be open-coded by reviewing all text line by line and then descriptive codes will be assigned to the words, sentences and paragraphs in the transcripts. At this stage of the analysis, lines in the transcripts will be linked and grouped as the first set of codes which relate to the current prescribing practices, the experiences of the ICU team, RA’s and champions, along with adaptations made. Axial coding in the next step of the data reduction process will link the descriptive codes via repackaging and combine the data to identify categories that have similar characteristics [65]. After developing categories, the relationships between the categories will be explored to reveal higher level themes, and then where relevant to implementation of the intervention mapped to the CFIR framework [64]. New themes, and contextual and team factors that emerge from the evaluation but do not fit the existing framework will also be reported.

## Discussion and potential impact

This study aims to deliver both local and generalised benefits. Locally, it will support implementation of a complex stewardship intervention in LMIC ICUs. By evaluating this process rigorously, it will build local capacity for further refinement and/or implementation of other complex interventions. If implementation is successful, and the intervention is effective, the project may deliver significant local benefits to ICUs including reducing costs, side-effects and antimicrobial resistance. Furthermore, implementation will be led by local stakeholders to give it the best chance of being sustainable beyond the duration of this study.

The study will also generate new generalisable knowledge. Firstly, it will describe pre-existing antimicrobial stewardship structures and processes in LMIC ICUs, permitting the identification and prioritisation of subsequent research and or improvement projects. Secondly, it will improve understanding about the efficacy of the proposed multifaceted implementation strategies in these settings. Thirdly, it will characterise the effectiveness of a widely-proposed but variably-implemented intervention which may deliver significant potential public health benefits.

## Limitations

The sample of participating ICUs is not proposed to be representative of the health systems in which they are situated. However, the range of settings that these ICUs represent is also a strength of the study; by clearly describing these contexts we will provide case studies which future research can build upon. We propose to mitigate this limitation on generalisability by rigorously describing their baseline structures and processes so that readers can infer how the study findings will apply to their own settings. The implementation strategies (particularly audit and feedback) require external facilitation and resources, further limiting generalisability. The quasi-experimental study design limits the attribution of causality to the implementation strategies and/or intervention. Finally, we will not follow-up implementation beyond month 7, limiting our knowledge of the sustainability of implementation beyond stakeholders’ perceptions.

## Supplementary Information

Supplementary Material 1. SPIRIT reporting checklist.

Supplementary Material 2. Site level survey.

Supplementary Material 3. Interview guide (months 4–6).

Supplementary Material 4. Interview guide (month 7). 

Supplementary Material 5. Participant Information Sheet.

Supplementary Material 6. Record of oral consent.

Supplementary Material 7. OxTrec minimal risk approval letter.

Supplementary Material 8. List of redundant combinations of antimicrobials.

## Data Availability

Data collected as part of this study protocol can be requested from the contact Principal Investigator, Duncan Wagstaff.

## Abbreviations

AMS: Antimicrobial Stewardship
AMR: Antimicrobial Resistance
ARI: Antimicrobial Resistance Index
DoT: Days of Therapy
AD: Antimicrobial Density
QI: Quality Improvement
CQR: Care Quality Registries
CCAA: Collaboration for Research Implementation, Training in Critical Care, Asia Africa
ARR: Antimicrobial Redundancy Rate
LMICs: Low- and Middle-Income Countries

## References

[1] Kaki, et al. Impact of antimicrobial stewardship in critical care: A systematic review. J Antimicrob Chemother. 2011;66(6):1223–30. 10.1093/jac/dkr137. 21460369 10.1093/jac/dkr137

[2] Organization, “Antimicrobial resistance : accelerating national and global responses,” 2024;3:3–8.

[3] Murray, et al. Global burden of bacterial antimicrobial resistance in 2019: a systematic analysis. Lancet. 2022;399(10325):629–55. 10.1016/S0140-6736(21)02724-0. 35065702 10.1016/S0140-6736(21)02724-0 PMC8841637

[4] O’Neill, “Tackling drug-resistant infections globally: Final report and recommendations,” 2016. 10.4103/2045-080x.186181.

[5] Chiotos, et al. Antibiotic stewardship in the intensive care unit: Challenges and opportunities. Infect Control Hosp Epidemiol. 2019;40(6):693–8. 10.1017/ice.2019.74.31046851 10.1017/ice.2019.74

[6] Wunderink, et al. Antibiotic Stewardship in the Intensive Care Unit. An Official American Thoracic Society Workshop Report in Collaboration with the AACN, CHEST, CDC, and SCCM. Ann Am Thorac Soc. 2020;17(5):531–40. 10.1513/AnnalsATS.202003-188ST. 32356696 10.1513/AnnalsATS.202003-188ST PMC7193806

[7] Ture et al., “Antimicrobial Stewardship in the Intensive Care Unit,” Antimicrob. Steward. Non-Traditional Settings a Pract. Guid., 2022;3:161–183 10.1007/978-3-031-21710-4_8.

[8] Thomas, et al. A Multicenter Evaluation of Prolonged Empiric Antibiotic Therapy in Adult ICUs in the United States. Crit Care Med. 2015;43(12):2527–34. 10.1097/CCM.0000000000001294. 26457751 10.1097/CCM.0000000000001294

[9] Quirós, et al. Antimicrobial stewardship programs in adult intensive care units in Latin America: Implementation, assessments, and impact on outcomes. Infect Control Hosp Epidemiol. 2022;43(2):181–90. 10.1017/ice.2021.80. 33829982 10.1017/ice.2021.80

[10] Marquet, et al. Incidence and outcome of inappropriate in-hospital empiric antibiotics for severe infection: a systematic review and meta-analysis. Crit Care. 2015;19(1):63. 10.1186/s13054-015-0795-y. 25888181 10.1186/s13054-015-0795-y PMC4358713

[11] World Health Organization, “Antimicrobial stewardship programmes in health-care facilities in low- and middle-income countries: a WHO practical toolkit,” 2019. 10.1093/jacamr/dlz072. 10.1093/jacamr/dlz072 PMC8210188 34222945

[12] Trivedi et al., “Opportunities to Improve Antibiotic Appropriateness in U.S. ICUs: A Multicenter Evaluation,” Crit. Care Med., vol. 48, no. 7, 2020. 10.1097/CCM.0000000000004344 32317600

[13] Thuy et al., “Hospital-acquired colonization and infections in a Vietnamese intensive care unit.,” PLoS One, vol. 13, no. 9, p. e0203600, 2018, 10.1371/journal.pone.0203600. 10.1371/journal.pone.0203600 PMC6128614 30192894

[14] McBride, et al. Catastrophic health care expenditure due to septic shock and dengue shock in Vietnam. Trans R Soc Trop Med Hyg. 2019;113(10):649–51. 10.1093/trstmh/trz064. 31340045 10.1093/trstmh/trz064 PMC6792161

[15] Pierce, et al. Global Antimicrobial Stewardship with a Focus on Low- and Middle-Income Countries. Int J Infect Dis. 2020;96:621–9. 10.1016/j.ijid.2020.05.126. 32505875 10.1016/j.ijid.2020.05.126 PMC7271868

[16] Hashimoto, et al. Impact of ward pharmacist-led antimicrobial stewardship in intensive care units. J Chemother. 2023;35(3):188–97. 10.1080/1120009X.2022.2087652. 35748502 10.1080/1120009X.2022.2087652

[17] Wu, et al. Barriers and facilitators of implementing interventions to improve appropriate antibiotic use in low- and middle-income countries: a systematic review based on the Consolidated Framework for Implementation Research. Implement Sci. 2022;17(1):30. 10.1186/s13012-022-01209-4. 35550169 10.1186/s13012-022-01209-4 PMC9096759

[18] Singh, et al. Investigating infection management and antimicrobial stewardship in surgery: a qualitative study from India and South Africa. Clin Microbiol Infect. 2021;27(10):1455–64. 10.1016/j.cmi.2020.12.013. 33422658 10.1016/j.cmi.2020.12.013

[19] Das et al., “The impact of training informal health care providers in India: A randomized controlled trial,” Science (80-. )., vol. 354, no. 6308, p. aaf7384, Oct. 2016, 10.1126/science.aaf7384. 10.1126/science.aaf7384 27846471

[20] Poluektova et al., “A scoping review and behavioural analysis of factors underlying overuse of antimicrobials.,” JAC-antimicrobial Resist., vol. 5, no. 3, p. dlad043, Jun. 2023, 10.1093/jacamr/dlad043. 10.1093/jacamr/dlad043 PMC10164659 37168837

[21] Kozlakidis et al., “Clinical Microbiology in Low Resource Settings.,” Frontiers in medicine, vol. 7. Switzerland, p. 258, 2020. 10.3389/fmed.2020.00258. 10.3389/fmed.2020.00258 PMC7297910 32587861

[22] Davey et al., “Interventions to improve antibiotic prescribing practices for hospital inpatients.,” Cochrane database Syst. Rev., vol. 2, no. 2, p. CD003543, Feb. 2017, 10.1002/14651858.CD003543.pub4. 10.1002/14651858.CD003543.pub4 PMC6464541 28178770

[23] Pollack, et al. Antibiotic Stewardship Programs in U.S. Acute Care Hospitals: Findings From the 2014 National Healthcare Safety Network Annual Hospital Survey. Clin Infect Dis. 2016;63(4):443–9. 10.1093/cid/ciw323. 27199462 10.1093/cid/ciw323 PMC6537894

[24] Howard, et al. An international cross-sectional survey of antimicrobial stewardship programmes in hospitals. J Antimicrob Chemother. 2015;70(4):1245–55. 10.1093/jac/dku497. 25527272 10.1093/jac/dku497

[25] O’Riordan, et al. Quality indicators for hospital antimicrobial stewardship programmes: a systematic review. J Antimicrob Chemother. 2021;76(6):1406–19. 10.1093/jac/dkab034. 33787876 10.1093/jac/dkab034

[26] Kotwani, et al. Prescriber and dispenser perceptions about antibiotic use in acute uncomplicated childhood diarrhea and upper respiratory tract infection in New Delhi: Qualitative study. Indian J Pharmacol. 2017;49(6):419–31. 10.4103/ijp.IJP_508_17. 29674796 10.4103/ijp.IJP_508_17 PMC5892023

[27] Chan, et al. SPIRIT 2013 explanation and elaboration: guidance for protocols of clinical trials. BMJ. 2013;346:1–42. 10.1136/bmj.e7586. 10.1136/bmj.e7586 PMC3541470 23303884

[28] Curran et al., “Effectiveness-implementation Hybrid Designs: Combining Elements of Clinical Effectiveness and Implementation Research to Enhance Public Health Impact,” Med. Care, vol. 50, no. 3, 2012. 10.1097/MLR.0b013e3182408812 PMC3731143 22310560

[29] Branch-Elliman, et al. Promoting de-implementation of inappropriate antimicrobial use in cardiac device procedures by expanding audit and feedback: protocol for hybrid III type effectiveness/implementation quasi-experimental study. Implement Sci. 2022;17(1):1–12. 10.1186/s13012-022-01186-8. 35093104 10.1186/s13012-022-01186-8 PMC8800400

[30] Becker, et al. Implementing contingency management for stimulant use in opioid treatment programs: protocol of a type III hybrid effectiveness-stepped-wedge trial. Implement Sci. 2023;18(1):1–15. 10.1186/s13012-023-01297-w. 37705093 10.1186/s13012-023-01297-w PMC10498624

[31] Yardley, et al. The Person-Based Approach to Intervention Development: Application to Digital Health-Related Behavior Change Interventions. J Med Internet Res. 2015;17(1): e30. 10.2196/jmir.4055. 25639757 10.2196/jmir.4055 PMC4327440

[32] Glasgow, et al. Evaluating the public health impact of health promotion interventions: the RE-AIM framework. Am J Public Health. 1999;89(9):1322–7. 10.2105/ajph.89.9.1322. 10474547 10.2105/ajph.89.9.1322 PMC1508772

[33] Glowacki, et al. Antibiotic Combinations with Redundant Antimicrobial Spectra: Clinical Epidemiology and Pilot Intervention of Computer-Assisted Surveillance. Clin Infect Dis. 2003;37(1):59–64. 10.1086/376623. 12830409 10.1086/376623

[34] Kim, et al. Redundant combinations of antianaerobic antimicrobials: impact of pharmacist-based prospective audit and feedback and prescription characteristics. Eur J Clin Microbiol Infect Dis. 2020;39(1):75–83. 10.1007/s10096-019-03687-9. 31482420 10.1007/s10096-019-03687-9

[35] Schultz, et al. Economic impact of redundant antimicrobial therapy in US hospitals. Infect Control Hosp Epidemiol. 2014;35(10):1229–35. 10.1086/678066. 25203175 10.1086/678066 PMC6487658

[36] Llewelyn, et al. Antibiotic review kit for hospitals (ARK-Hospital): a stepped-wedge cluster-randomised controlled trial. Lancet Infect Dis. 2023;23(2):207–21. 10.1016/S1473-3099(22)00508-4. 36206793 10.1016/S1473-3099(22)00508-4

[37] Tamma, et al. Rethinking How Antibiotics Are Prescribed: Incorporating the 4 Moments of Antibiotic Decision Making Into Clinical Practice. JAMA. 2019;321(2):139–40. 10.1001/jama.2018.19509. 30589917 10.1001/jama.2018.19509

[38] Haque et al., “Impact of pharmacist-led antibiotic stewardship program in a PICU of low/middle-income country.,” BMJ open Qual., vol. 7, no. 1, p. e000180, 2018, 10.1136/bmjoq-2017-000180. 10.1136/bmjoq-2017-000180 PMC5759741 29333498

[39] Wagstaff et al., “Interventions for improving critical care in Low- and Middle-Income Countries: a systematic review.,” Intensive Care Med., vol. In press. 10.1007/s00134-024-07377-9 38748264

[40] Grimshaw et al., “Effectiveness and efficiency of guideline dissemination and implementation strategies.,” Health Technol. Assess., vol. 8, no. 6, pp. iii–iv, 1–72, Feb. 2004, 10.3310/hta8060. 10.3310/hta8060 14960256

[41] Pisani et al., “Performance evaluation of a multinational data platform for critical care in Asia,” Wellcome Open Res., vol. 6, no. 251, 2022, 10.12688/wellcomeopenres.17122.2. 10.12688/wellcomeopenres.17122.1 PMC8812332 35141427

[42] Beane, et al. Establishing a critical care network in Asia to improve care for critically ill patients in low- and middle-income countries. Crit Care. 2020;24(1):608. 10.1186/s13054-020-03321-7. 33059761 10.1186/s13054-020-03321-7 PMC7558669

[43] Pulcini, et al. Design of a ‘day 3 bundle’ to improve the reassessment of inpatient empirical antibiotic prescriptions. J Antimicrob Chemother. 2008;61(6):1384–8. 10.1093/jac/dkn113. 18367462 10.1093/jac/dkn113

[44] Ivers et al., “Audit and feedback : effects on professional practice and healthcare outcomes ( Review ),” Cochrane database Syst. Rev., vol. 6, no. 6, p. CD000259, 2012, 10.1002/14651858.CD000259.pub3.www.cochranelibrary.com. 10.1002/14651858.CD000259.pub3 PMC11338587 22696318

[45] Salluh, et al. National ICU Registries as Enablers of Clinical Research and Quality Improvement. Crit Care Med. 2024;52(1):125–35. 10.1097/CCM.0000000000006050. 37698452 10.1097/CCM.0000000000006050

[46] Mwangi et al., “Organisation, staffing and resources of critical care units in Kenya.,” PLoS One, vol. 18, no. 7, p. e0284245, 2023, 10.1371/journal.pone.0284245. 10.1371/journal.pone.0284245 PMC10374136 37498872

[47] Satterfield, et al. The role of education in antimicrobial stewardship. J Hosp Infect. 2020;105(2):130–41. 10.1016/j.jhin.2020.03.028. 32243953 10.1016/j.jhin.2020.03.028

[48] Aryal et al., “Feasibility and acceptability of implementing a practice guideline for the use of high flow nasal cannula in critically ill patients who have hypoxemia: a multi-centre study in Nepal,” Wellcome Open Res., vol. 8, no. 196, 2023, 10.12688/wellcomeopenres.19223.1.

[49] Tripathy et al., “AIIMS ICU Rehabilitation (AIR): development and description of intervention for home rehabilitation of chronically ill tracheostomized patients,” Wellcome Open Res., vol. 8, no. 285, 2024, 10.12688/wellcomeopenres.19340.2. 10.12688/wellcomeopenres.19340.4 PMC11399758 39280064

[50] A. et al., “A learning health systems approach to improving the quality of care for patients in South Asia,” Glob. Health Action, vol. 12, no. 1, p. 1587893, 2019, 10.1080/16549716.2019.1587893 PT - Article. 10.1080/16549716.2019.1587893 PMC6461109 30950778

[51] Damschroder, et al. Conceptualizing outcomes for use with the Consolidated Framework for Implementation Research (CFIR): the CFIR Outcomes Addendum. Implement Sci. 2022;17(1):7. 10.1186/s13012-021-01181-5. 35065675 10.1186/s13012-021-01181-5 PMC8783408

[52] Harris, et al. Research electronic data capture (REDCap)–a metadata-driven methodology and workflow process for providing translational research informatics support. J Biomed Inform. 2009;42(2):377–81. 10.1016/j.jbi.2008.08.010. 18929686 10.1016/j.jbi.2008.08.010 PMC2700030

[53] Harris, et al. The REDCap consortium: Building an international community of software platform partners. J Biomed Inform. 2019;95: 103208. 10.1016/j.jbi.2019.103208. 31078660 10.1016/j.jbi.2019.103208 PMC7254481

[54] Ahuja, et al. An evaluation of the implementation of interventions to reduce postoperative infections and optimise antibiotic use across the surgical pathway in India: a mixed-methods exploratory study protocol. Pilot Feasibility Stud. 2022;8(1):237. 10.1186/s40814-022-01192-z. 36335367 10.1186/s40814-022-01192-z PMC9636821

[55] Pisani et al., “Performance evaluation of a multinational data platform for critical care in Asia,” Wellcome Open Res., vol. 6, 2021, 10.12688/wellcomeopenres.17122.1. 10.12688/wellcomeopenres.17122.1 PMC8812332 35141427

[56] “The TRUST Code – A Global Code of Conduct for Equitable Research Partnerships.”

[57] University of Oxford, “OxTREC.” https://researchsupport.admin.ox.ac.uk/governance/ethics/apply/oxtrec

[58] Charif et al., “Effective strategies for scaling up evidence- based practices in primary care : a systematic review,” pp. 1–13, 2017, 10.1186/s13012-017-0672-y. 10.1186/s13012-017-0672-y PMC5700621 29166911

[59] Durlak et al., “Implementation Matters : A Review of Research on the Influence of Implementation on Program Outcomes and the Factors Affecting Implementation,” pp. 327–350, 2008, 10.1007/s10464-008-9165-0. 10.1007/s10464-008-9165-0 18322790

[60] An, et al. What Really Works in Intervention? Using Fidelity Measures to Support Optimal Outcomes. Phys Ther. 2020;100(5):757–65. 10.1093/ptj/pzaa006. 31944249 10.1093/ptj/pzaa006

[61] Guerbaai et al., “Evaluating the implementation fidelity to a successful nurse-led model ( INTERCARE ) which reduced nursing home unplanned hospitalisations,” vol. 8, pp. 1–17, 2023. 10.1186/s12913-023-09146-8 PMC9910256 36759902

[62] Spee et al., “Establishing Treatment Fidelity in Evidence-Based Parent Training Programs for Externalizing Disorders in Children and Adolescents,” pp. 230–247, 2014, 10.1007/s10567-014-0166-2. 10.1007/s10567-014-0166-2 24706293

[63] Eiraldi et al., “Implementation fidelity , student outcomes , and cost ‑ effectiveness of train ‑ the ‑ trainer strategies for Masters ‑ level therapists in urban schools : results from a cluster randomized trial,” Implement. Sci., pp. 1–19, 2024, 10.1186/s13012-023-01333-9. 10.1186/s13012-023-01333-9 PMC10809609 38273369

[64] “Consolidated Framework for Implementation Research.” https://cfirguide.org/

[65] Sheron, et al. Healthcare provider and patient perspectives on access to and management of atrial fibrillation in the Northern Province, Sri Lanka: a rapid evaluation of barriers and facilitators to care. BMC Health Serv Res. 2022;22(1):1–13. 10.1186/s12913-022-08440-1. 35999563 10.1186/s12913-022-08440-1 PMC9400248

