# Correction: Evidence based QUality Improvement for Prescribing Stewardship in ICU (EQUIPS-ICU): protocol for type III hybrid implementation-effectiveness study

**DOI:** 10.1186/s13012-025-01425-8

**Published:** 2025-03-31

**Authors:** Duncan Wagstaff, John Amuasi, Sumaiya Arfin, Diptesh Aryal, Mohd Basri Mat Nor, Joseph Bonney, Arjen Dondorp, David Dongelmans, Layoni Dullawe, Fathima Fazla, Aniruddha Ghose, Eva Hanciles, Rashan Haniffa, Madiha Hashmi, Adam Hewitt Smith, Bharath Kumar, Yen Lam Minh, Ramani Moonesinghe, Luigi Pisani, Cornelius Sendagire, Mohd Shahnaz Hasan, Maryam Shamal Ghalib, Moses Siaw Frimpong, Otavio Ranzani, Menbeu Sultan, David Thomson, Swagata Tripathy, Louise Thwaites, Rabiul Alam Md. Erfan Uddin, Mohd Zulfakar Mazlan, Wangari Waweru-Siika, Abigail Beane

**Affiliations:** 1 Centre for Preoperative Medicine, University College London, London, UK; 2 Department of Global Health, Kwame Nkrumah University of Science and Technology, Kumasi, Ghana; 3 The George Institute for Global Health, New Delhi, India; 4 Amsterdam Public Health (APH), Amsterdam University Medical Centre(UMC), Amsterdam, The Netherlands; 5 Mahidol Oxford Tropical Medicine Research Unit, Bangkok, Thailand; 6 Department of Critical Care, Nepal Intensive Care Research Foundation, Kathmandu, Nepal; 7 Department of Intensive Care Anaesthesiology, International Islamic University Malaysia, Kuala Lumpur, Malaysia; 8 Emergency Medicine Department, Komfo Anokye Teaching Hospital, Kumasi, Ghana; 9 Amsterdam Institute for Global Health and Development, Amsterdam, The Netherlands; 10 Nufeld Department of Medicine, University of Oxford, Oxford, UK; 11 National Intensive Care Evaluation (NICE) Foundation, Amsterdam, The Netherlands; 12 Department of Intensive Care Medicine, UMC, University of Amsterdam, Amsterdam, The Netherlands; 13 Nat-Intensive Care Surveillance, Mahidol Oxford Tropical Medicine Research Unit, Colombo, Sri Lanka; 14 Department of Medicine, Chittagong Medical College Hospital, Chattogram, Bangladesh; 15 Connaught Hospital, Freetown, Sierra Leone; 16 Ministry of Health and Sanitation, Free- town, Sierra Leone; 17 Pandemic Sciences Hub and Institute for Regeneration and Repair, University of Edinburgh, Edinburgh, UK; 18 Department of Critical Care Medicine, Ziauddin University, Karachi, Pakistan; 19 Faculty of Health Sciences, Busitema University, Mbale, Uganda; 20 William Harvey Research Institute, Queen Mary University of London, London, UK; 21 Department of Critical Care Medicine, Apollo Hospitals Educational and Research Foundation, Chennai, India; 22 Clinical Research Unit, Oxford University, University of Oxford, Ho Chi Minh City, Vietnam; 23 D’Or Institute for Research and Education, Sao Paulo, Brazil; 24 Uganda Heart Institute, University of Makerere, Makerere, Uganda; 25 General Surgery, Wazir Akbar Khan Hospital, Kabul, Afghanistan; 26 Department of Anaesthesiology and Intensive Care, Komfo Anokye Teaching Hospital, Kumasi, Ghana; 27 Barcelona Institute for Global Health, ISGlobal, Barcelona, Spain; 28 Pulmonary Division, Heart Institute, Faculty of Medicine, Hospital das Clínicas da Faculdade de Medicina da Universidade de São Paulo, São Paulo, Brazil; 29 Department of Emergency Medicine and Critical Care, St. Paul’s Hospital Millennium Medical College, Addis Ababa, Ethiopia; 30 Department of Anaesthesia and Perioperative Medicine, University of Cape Town, Cape Town, South Africa; 31 AII India Institute of Medical Sciences, New Delhi, India; 32 Hospital Universiti Sains Malaysia, Kota Bharu, Malaysia; 33 Department of Anaesthesia, The Aga Khan University, Nairobi, Kenya; 34 Department of Implementation Research, Bernhard Nocht Institute of Tropical Medicine, Hamburg, Germany; 35 Global Health and Infectious Disease Research Group, KCCR, KNUST, Kumasi, Ghana

**Correction** **:** **Implementation Sci 20, 12 (2025)**

**https://doi.org/10.1186/s13012-024-01413-4**

Following the publication of the original article [1], the authors reported that the name of the 2^nd^ author was misspelled.

**Incorrect**

John Amausi

**Correct**

John Amuasi

John Amuasi is affiliated with affiliation number 2, as well as Department of Implementation Research, Bernhard Nocht Institute of Tropical Medicine, Hamburg, Germany. This affiliation has been added as Affiliation no. 34, and Global Health and Infectious Disease Research Group, KCCR, KNUST, Kumasi, Ghana (Affiliation no. 35)

Furthermore, the affiliation of Joseph Bonney was incorrect. Instead of Virology Department, Noguchi Memorial Institute for Medical Research, Accra, Ghana, Bonney’s affiliations are as follows: 

Emergency Medicine Department, Komfo Anokye Teaching Hospital, Kumasi, Ghana (Affiliation no. 8) and Global Health and Infectious Disease Research Group, KCCR, KNUST, Kumasi, Ghana (Affiliation no. 35).

This correction shows the correct names and affiliations for all authors. The original article has been corrected.
