# Supplementary material for: Evidence based QUality Improvement for Prescribing Stewardship in ICU (EQUIPS-ICU): protocol for type III hybrid implementation-effectiveness study
Source: Implement Sci. 2025 Feb 25;20:12. doi: 10.1186/s13012-024-01413-4 (PMC11863957; doi:10.1186/s13012-024-01413-4)
Supplement: Supplementary file 2 — Supplementary Material 2. Site level survey. [file 13012_2024_1413_MOESM2_ESM.pdf]

Site-level survey of antimicrobial stewardship core elements

About the survey:
This survey consists of * questions broken into 6 sections: Leadership commitment, Accountability and Responsibility, AMS actions, Education and Training, Monitoring and Surveillance, Reporting and Feedback. This survey will be **live for 4 weeks;** closing date 1 December 2024*.

How will we use your information? The findings from this survey may be published in a report, a scientific journal, presented at a conference and made openly available to the public. Participation is voluntary. **Your identity will remain anonymous** in all publications, presentations and public releases. Your identity will only be requested if you give permission for us to keep in contact about this project and if you specify that you wish to be considered for invitation to future surveys. **Your identity and contact details will not be shared beyond the research team working directly on this project and will be removed prior to the analysis of the survey findings.** The information that you supply will be treated in accordance with the University of Oxford's Data Protection Policy. This project has been reviewed and approved as a minimal risk project by the Oxford Tropical Research and Ethics Committee (OxTREC). **Reference: EQUIPS_ Site-level survey_version 2.0 dated 20 August 2024. OxTREC *(559-24).***

To complete this self-assessment, please provide one response per question based on the following options:
1. **No**- the core element is not in place and is not a priority.
2. **No, but a priority** – the core element is a priority but there is no plan in place to initiate it.
3. **Planned but not started** – the core element is planned but no action has taken place.
4. **Partially implemented** – the core element is in place, but it is only partially implemented requiring further strengthening.
5. **Fully implemented** – the core element is in place and is fully implemented without requiring strengthening but needing to be sustained.

Only one survey should be completed for each ICU. Please liaise with your local colleagues if there are questions you cannot answer.

* Indicates required question

Agreement to participate

List of abbreviations/definitions

ICU	Intensive Care Unit
AMS	Antimicrobial Stewardship
PPS	Point prevalence study
IPC	Infection Prevention and Control
Antibiogram	Profile of antimicrobial susceptibilities of organisms isolated from patients within an institution

1. **Clicking on the "I Agree to participate" button indicates that:** *

• I understand that my participation in this survey is **completely voluntary;** there are no right or wrong answers.

•I may *end the survey at any time* without consequence.

•There are no known risks associated with this study and all information will be kept **confidential.**

•I am **over the age of 18.**

•I have **read and understood** all the above information about the survey.

Mark only one

Check all that apply.

- [ ] I Agree
- [ ] I Disagree

2. Name of the hospital *

____

Skip to question 3

Leadership Commitment

3. 1. Is AMS identified as a priority by the ICU management/leadership? *

Suggested verifiers: Interviews with ICU leadership

Mark only one oval.

- No
- No, but a priority
- Planned but not started
- Partially implemented
- Fully implemented
- Don't know

4. 1.a. Are AMS activities included in the ICU's annual plans with key performance indicators? *

Suggested verifiers: Annual plans of the ICU with AMS performance indicators

Mark only one oval.

- No
- No, but a priority
- Planned but not started
- Partially implemented
- Fully implemented
- Don't know

5. 1.b. Has the ICU management allocated human and financial resources to initiate AMS activties? *

Suggested verifiers: Availability of personnel with AMS role in their job description

Mark only one oval.

- No
- No, but a priority
- Planned but not started
- Partially implemented
- Fully implemented
- Don't know

6. 2. Is there an ICU level action plan in place that prioritises AMS activities? *

Suggested verifiers: ICU level action plan and AMS progress report

Mark only one oval.

- No
- No, but a priority
- Planned but not started
- Partially implemented
- Fully implemented
- Don't know

7. 2.a. Is there a mechanism to regularly monitor and measure the implementation of AMS activities in the ICU? *

Suggested verifiers: Annual/quarterly progress report on the implementation of the ICU level AMS action plan

Mark only one oval.

- No
- No, but a priority
- Planned but not started
- Partially implemented
- Fully implemented
- Don't know

8. 3. Is there dedicated financial support for the ICU-level AMS action plan? *

Suggested verifiers: ICU budget line with dedicated funds for AMS

Mark only one oval.

- No
- No, but a priority
- Planned but not started
- Partially implemented
- Fully implemented
- Don't know

9. 3.a. Has a budget (e.g.annual) for the implementation of the ICU AMS action plan been developed? *

Suggested verifiers: Developed ICU level budget for AMS

Mark only one oval.

- No
- No, but a priority
- Planned but not started
- Partially implemented
- Fully implemented
- Don't know

Accountability and Responsibility

10. 4. Is there a multidisciplinary AMS committee leadership in the ICU with clear terms of reference (TOR)? *

Suggested verifiers: AMS committee TOR

Mark only one oval.

- No
- No, but a priority
- Planned but not started
- Partially implemented
- Fully implemented
- Don't know

11. 4.a. Does the AMS committee/other relevant committee meet on a regular basis (minimum monthly or quarterly)? *

Suggested verifiers: Minutes of last AMS committee meeting

Mark only one oval.

- No
- No, but a priority
- Planned but not started
- Partially implemented
- Fully implemented
- Don't know

12. 5. Is there a dedicated AMS leader/ champion identified for the ICU? *

Suggested verifiers: AMS champion identified

Mark only one oval.

- No
- No, but a priority
- Planned but not started
- Partially implemented
- Fully implemented
- Don't know

13. 5.a. Does the team leader or champion have dedicated staff time for AMS activity in their TOR/job description? *

Suggested verifiers: TOR/job description includes AMS activities

Mark only one oval.

- No
- No, but a priority
- Planned but not started
- Partially implemented
- Fully implemented
- Don't know

14. 6. Is there an AMS team for the ICU with clear terms of reference? *

Suggested verifiers: AMS team TOR

Mark only one oval.

- No
- No, but a priority
- Planned but not started
- Partially implemented
- Fully implemented
- Don't know

15. 6.a. Does the AMS team meet on a regular basis? *

Suggested verifiers: Last AMS team meeting minutes (minimum weekly)

Mark only one oval.

- No
- No, but a priority
- Planned but not started
- Partially implemented
- Fully implemented
- Don't know

16. 7. Are other ICU health-care professionals apart from the AMS team involved in AMS activities? *

Suggested verifiers: Evidence of involvement of other health-care professionals in AMS activities

Mark only one oval.

- No
- No, but a priority
- Planned but not started
- Partially implemented
- Fully implemented
- Don't know

17. 8. Does the AMS committee/team collaborate with other health-care teams such as drug and therapeutics, infection prevention and control (IPC), HIV/tuberculosis (TB) or quality improvement? *

Suggested verifiers: Evidence to show collaboration or joint meetings

Mark only one oval.

- No
- No, but a priority
- Planned but not started
- Partially implemented
- Fully implemented
- Don't know

18. 9. Does the AMS team/committee produce regular (descriptive) activity reports on the implementation of the AMS programme in the ICU? *

Suggested verifiers: AMS committee report

Mark only one oval.

- No
- No, but a priority
- Planned but not started
- Partially implemented
- Fully implemented
- Don't know

19. 9.a. Is the AMS activity report disseminated to the ICU management, other ICU team members and appropriate hospital/national authority? *

Suggested verifiers: Latest AMS activity report, summary report to national AMS technical working group

Mark only one oval.

- No
- No, but a priority
- Planned but not started
- Partially implemented
- Fully implemented
- Don't know

AMS actions

20. 10. Is there a standard treatment guideline at the ICU? *

Suggested verifiers: ICU standard treatment guideline, including infection prevention management guidelines

Mark only one oval.

- No
- No, but a priority
- Planned but not started
- Partially implemented
- Fully implemented
- Don't know

21. 10.a. Are the guidelines reviewed and updated periodically based on the availability of new evidence? *

Suggested verifiers: Documentation of guideline review processes

Mark only one oval.

- No
- No, but a priority
- Planned but not started
- Partially implemented
- Fully implemented
- Don't know

22. 11. Is there a regular review of antimicrobial prescriptions at the ICU? *

Suggested verifiers: AMS audit report

Mark only one oval.

- No
- No, but a priority
- Planned but not started
- Partially implemented
- Fully implemented
- Don't know

23. 11.a. Which of these components of prescriptions are explicitly reviewed? Please select all that apply. *

Mark only one oval.

- Indication
- Route of antimicrobial (e.g., enteral vs parenteral)
- Duration
- Stop date
- Other

24. 11.a. (i) If Other, please specify

____

25. 11.b. Who performs this review? *

Mark only one oval.

- ICU doctors
- ICU nurses
- Microbiology/Infectious disease
- Pharmacy
- Other

26. 11.b.(i) If Other, please specify

____

27. 11.c. When does the review happen in the ICU working day? *

Check all that apply.

- [ ] During the ICU ward round
- [ ] On a dedicated AMS ward round
- [ ] Other

28. 11.c. (i) If other, please specify.

____

29. 11.d. How often does the review take place? *

Mark only one oval.

- Daily
- More frequently than daily, less than weekly
- Weekly
- Less frequently than weekly

30. 11.e. How is the review documented? *

Mark only one oval.

- On the drug chart
- In the patient's notes
- On a dedicated review form
- Other

31. 11.e.(i) If other, please specify *

____

32. 11.f. Is there a written protocol describing how the review should be conducted? *

Check all that apply.

- [ ] Yes
- [ ] No

33. 12. Is the advice/feedback from AMS teams easily accessible /available to prescribers in the ICU? *

Suggested verifiers: Feedback report from AMS team

Mark only one oval.

- No
- No, but a priority
- Planned but not started
- Partially implemented
- Fully implemented
- Don't know

34. 13. Does the AMS team conduct regular ward rounds and other AMS interventions in the ICU? *

Suggested verifiers: Ward round reports

Mark only one oval.

- No
- No, but a priority
- Planned but not started
- Partially implemented
- Fully implemented
- Don't know

35. 14. Does the ICU have a formulary/list of approved antibiotics for use based on the national formulary? *

Suggested verifiers: ICU formulary/drug bulletin report

Mark only one oval.

- No
- No, but a priority
- Planned but not started
- Partially implemented
- Fully implemented
- Don't know

36. 14.a. Does the ICU formulary specify lists of restricted antibiotics that require approval by a designated team or person (pre-authorization)? *

Suggested verifiers: ICU formulary with restrictions

Mark only one oval.

- No
- No, but a priority
- Planned but not started
- Partially implemented
- Fully implemented
- Don't know

37. 15. Does the ICU have access to laboratory and imaging services (on-site or off-site) that can be used to support AMS interventions? *

Suggested verifiers: Sample laboratory report

Mark only one oval.

- No
- No, but a priority
- Planned but not started
- Partially implemented
- Fully implemented
- Don't know

38. 16. Are there information technology (IT) services, tally cards or other inventory control tools available that can be used to support data gathering to support AMS activities? *

Suggested verifiers: Availability of functional IT services, inventory control tools

Mark only one oval.

- No
- No, but a priority
- Planned but not started
- Partially implemented
- Fully implemented
- Don't know

39. 17. Are there standardized prescription charts, medical records/ patient folders and transfer notes to support treatment and AMS activities in the ICU? *

Suggested verifiers: Availability of prescription charts and medical records

Mark only one oval.

- No
- No, but a priority
- Planned but not started
- Partially implemented
- Fully implemented
- Don't know

40. 17.a. Does the ICU have a written policy that requires prescribers to document the indication and antibiotics prescribed in a prescription chart/medical records? *

Suggested verifiers: Policies on prescribing

Mark only one oval.

- No
- No, but a priority
- Planned but not started
- Partially implemented
- Fully implemented
- Don't know

Education and training

41. 18. Does the ICU include AMS programmes such as optimising antibiotic prescribing, dispensing and administration in the staff induction training? *

Suggested verifiers: Induction training manuals

Mark only one oval.

- No
- No, but a priority
- Planned but not started
- Partially implemented
- Fully implemented
- Don't know

42. 19. Does the ICU offer continuous in-service training or continuous professional development on AMS, infection prevention and control (IPC) to staff? *

Suggested verifiers: In-service training manuals/continuous professional development/continuous medical education

Mark only one oval.

- No
- No, but a priority
- Planned but not started
- Partially implemented
- Fully implemented
- Don't know

43. 20. Does the ICU ensure training for the AMS team on AMS/ IPC? *

Suggested verifiers: Training reports, interviews with staff

Mark only one oval.

- No
- No, but a priority
- Planned but not started
- Partially implemented
- Fully implemented
- Don't know

Monitoring and surveillance

44. 21. Are regular prescription audits, point prevalence surveys to assess the appropriateness of antibiotic prescribing undertaken at the ICU by the AMS committee or relevant team? *

Suggested verifiers: Number of audits conducted with report

Mark only one oval.

- No
- No, but a priority
- Planned but not started
- Partially implemented
- Fully implemented
- Don't know

45. 22. Does the ICU regularly monitor the quantity and types of antibiotic use (purchased/ prescribed/ dispensed)? *

Suggested verifiers: Antimicrobial consumption report

Mark only one oval.

- No
- No, but a priority
- Planned but not started
- Partially implemented
- Fully implemented
- Don't know

46. 22.a. Does the ICU regularly monitor shortages/stockouts of essential antimicrobials? *

Suggested verifiers: Stock-out report

Mark only one oval.

- No
- No, but a priority
- Planned but not started
- Partially implemented
- Fully implemented
- Don't know

47. 22.b. Is there a mechanism to report substandard and falsified medicines and diagnostics at the ICU? *

Suggested verifiers: Reports of substandard/ falsified antimicrobials and diagnostics

Mark only one oval.

- No
- No, but a priority
- Planned but not started
- Partially implemented
- Fully implemented
- Don't know

48. 23. Does the AMS team regularly monitor antibiotic susceptibility and resistance rates for a range of key indicator bacteria? *

Suggested verifiers: Antimicrobial surveillance report

Mark only one oval.

- No
- No, but a priority
- Planned but not started
- Partially implemented
- Fully implemented
- Don't know

49. 24. Does the AMS team monitor compliance with at least one specific AMS intervention (e.g. indication captured in medical records for patients) at the ICU? *

Suggested verifiers: AMS intervention report

Mark only one oval.

- No
- No, but a priority
- Planned but not started
- Partially implemented
- Fully implemented
- Don't know

Reporting feedback within the ICU

50. 25. Does the AMS committee/ relevant team analyse and report on the quantities of antibiotics purchased/ prescribed/ dispensed to prescribers and ICU management? *

Suggested verifiers: Antimicrobial consumption report

Mark only one oval.

- No
- No, but a priority
- Planned but not started
- Partially implemented
- Fully implemented
- Don't know

51. 26. Does the AMS committee/ relevant team review, analyse and report on antibiotic susceptibility rates and key findings shared with prescribers? *

Suggested verifiers: Evidence of dissemination of susceptibility report

Mark only one oval.

- No
- No, but a priority
- Planned but not started
- Partially implemented
- Fully implemented
- Don't know

52. 27. Does the AMS team communicate findings from audits/reviews of the quality/appropriateness of antibiotic use to ICU prescribers along with specific action points? *

Suggested verifiers: Reports to prescribers with action points

Mark only one oval.

- No
- No, but a priority
- Planned but not started
- Partially implemented
- Fully implemented
- Don't know

53. 28. Does the ICU develop and aggregate antibiograms (Annex VIII, page 71 of the WHO AMS toolkit) and regularly update it? *

Suggested verifiers: Availability of antibiogram

Mark only one oval.

- No
- No, but a priority
- Planned but not started
- Partially implemented
- Fully implemented
- Don't know

54. 28.a. If antibiogram is published, please share citation below. *

____

This content is neither created nor endorsed by Google.

Google Forms
