# Supplementary material for: Evidence based QUality Improvement for Prescribing Stewardship in ICU (EQUIPS-ICU): protocol for type III hybrid implementation-effectiveness study
Source: Implement Sci. 2025 Feb 25;20:12. doi: 10.1186/s13012-024-01413-4 (PMC11863957; doi:10.1186/s13012-024-01413-4)
Supplement: Supplementary file 3 — Supplementary Material 3. Interview guide (months 4–6). [file 13012_2024_1413_MOESM3_ESM.docx]

**Supplementary Material**

**Appendix two**

**Interview Guide (months 4-6)**

*For semi-structured remote interviews with site Champions during months 4-6*

**Study Title:** Evidence based QUality Improvement for Prescribing Stewardship in ICU (EQUIPS-ICU). Can a structured antimicrobial review be implemented in LMIC ICUs?

**Interviewer guidance**

Introduce yourself

Explain the Participant Information Sheet to the participant before the interview and answer any questions they may have. Ensure they understand the interview will be recorded. Ask this questions after the explanation to confirm participation:

● **Do you agree to take part?** Commence only once the participant agrees to this.

The interview will be semi-structured in nature, and the questions can evolve in the context of the conversation, but the broad outline is as follows:

**Champion details**

1. Please clarify your experience & role in the ICU/hospital
   1. Clinical role
   2. Management role
   3. Time at that ICU
   4. Prior AMS experience
   5. Prior QI experience

**Fidelity:**

1. Who is expected to conduct your antimicrobial review? Who is actually conducting it?
2. When is the review expected to happen during the ICU day? When does it actually happen?
3. Where is the review expected to take place? Where does it actually happen?
4. How frequently are you expecting to conduct reviews? (e.g. twice-daily? Daily? weekly?) How frequently are they actually happening?
5. How are the review conclusions documented and communicated? How are they actually documented and communicated?
6. Which professional roles (e.g. microbiology, infectious disease, pharmacy etc) are *intended* to be involved in the review process? Which roles are *actually* involved? What is the *impact* of their involvement?
7. In what ways has your review process changed since before the project began? What has been involved in aligning the new review process with existing ICU structures?
8. What are the most common reasons for inappropriate prescribing? How are you addressing these?
   1. Indication
   2. Route
   3. Duration
   4. Stop date
   5. other
9. Have you written a protocol for the review? How has it been shared?
10. Audit & Feedback
    1. Who did you plan to collect the data for the project CRF? Who is actually collecting it?
    2. When do they collect the data?
    3. What changes to data collection processes do you plan?
    4. How have you been sharing feedback? (when/where/format/how frequently)
    5. How many sessions did you plan? How many have you had?
    6. Who is your intended audience? Who is the actual audience?
    7. What topics have you discussed? What were the conclusions of these discussions?
    8. How do you present the feedback (e.g. verbally? In writing? Are reports shared?).
11. Education platform and material:
    1. Which of your team members are you intending to access it? Who has accessed it so far?
    2. How has it influenced this project? Or wider activities?
    3. What changes would you like to see? (e.g. accessibility / amount of content / focus of content)

**Reach**

1. How did you expect to identify patients for review? How are they actually being identified?
2. Which patients are you expecting to review? Which patients are you actually reviewing?
3. Are there any particular groups of patients you are struggling to review or need to conduct the review differently? e.g.
   1. Surgical patients
   2. Patients admitted with pre-existing antimicrobial prescriptions?
   3. Early discharges

**Adaptations**

1. What adaptations have been made?
   1. Protocol
   2. A&F:
      1. Data collection
      2. Feedback
   3. Education
   4. Other (e.g. posters, patient involvement strategies…)

**Adoption**

1. What evidence do you have that the review process will be continued in month 7?
2. What would you do now to ensure it does continue in month 7?

**Sustainability**

1. What evidence do you have that the review will be sustained in the long term?
2. What needs to happen now to make the review process sustainability?
