# Supplementary material for: Evidence based QUality Improvement for Prescribing Stewardship in ICU (EQUIPS-ICU): protocol for type III hybrid implementation-effectiveness study
Source: Implement Sci. 2025 Feb 25;20:12. doi: 10.1186/s13012-024-01413-4 (PMC11863957; doi:10.1186/s13012-024-01413-4)
Supplement: Supplementary file 4 — Supplementary Material 4. Interview guide (month 7). [file 13012_2024_1413_MOESM4_ESM.docx]

**Supplementary Material**

**Appendix three**

**Interview Guide (month 7)**

*For semi-structured remote interviews with site Champions during month 7*

**Study Title:** Evidence based QUality Improvement for Prescribing Stewardship in ICU (EQUIPS-ICU). Can a structured antimicrobial review be implemented in LMIC ICUs?

**Interviewer guidance**

Introduce yourself

Explain the Participant Information Sheet to the participant before the interview and answer any questions they may have. Ensure they understand the interview will be recorded. Ask this questions after the explanation to confirm participation:

● **Do you agree to take part?** Commence only once the participant agrees to this.

The interview will be semi-structured in nature, and the questions can evolve in the context of the conversation. To be adapted in light of findings from baseline interviews and quantitative evaluation of implementation but the broad outline is as follows:

**Adoption:**

1. Are you still conducting antimicrobial reviews?
   1. If not, why not?
   2. If yes, who made the decision to continue, and for what reasons?

**Fidelity:**

1. Who is conducting your antimicrobial review?
2. When does it happen during the working day?
3. Where does it take place?
4. How frequently does it take place?
5. How are conclusions documented and communicated?
6. Is the protocol still active?
7. Which data are being collected to describe conduct of review or antimicrobial prescribing?
8. Which data are fed back to local prescribers? When/where/which format/how frequently?
9. Which audience receives the feedback?
10. Which topics have you discussed? What actions have been taken as a results of the feedback? What are the impacts of those actions?
11. Who has accessed the education platform? Were there any differences in who accessed the platform to what you were expecting?
12. What changes to the platform would you like to see?

**Adaptations**

1. What adaptations have been made?
   1. Protocol
   2. A&F:
      1. Data collection
      2. Feedback
   3. Education
   4. Other

**Sustainability**

1. What evidence do you have that the review process will be sustained beyond month 8?
   1. What further adaptations would help it be sustained locally?
