# Supplementary material for: Evidence based QUality Improvement for Prescribing Stewardship in ICU (EQUIPS-ICU): protocol for type III hybrid implementation-effectiveness study
Source: Implement Sci. 2025 Feb 25;20:12. doi: 10.1186/s13012-024-01413-4 (PMC11863957; doi:10.1186/s13012-024-01413-4)
Supplement: Supplementary file 5 — Supplementary Material 5. Participant Information Sheet. [file 13012_2024_1413_MOESM5_ESM.docx]

**PARTICIPANT INFORMATION SHEET**

***We will give you information about the study to help you decide whether or not you will agree to participate. If you have any questions or concerns, you will have a chance to discuss them with the study staff.***

**Study Title:** Evidence based QUality Improvement for Prescribing Stewardship in ICU

(EQUIPS- ICU). Can a structured antimicrobial review be implemented in LMIC

ICUs?

**Short Title:** Implementing a structured antimicrobial review in LMIC ICUs

**Investigators:** Dr Duncan Wagstaff, Dr Abi Beane

**Who are the researchers and what is the purpose?**

The study is being conducted by the Critical Care Asia-Africa (CCAA). The purpose of the study is to find out if we can introduce a structured antimicrobial review in ICUs. Today, you are being invited to take part because you are healthcare workers coordinating implementation in the Intensive Care Units.

**What will happen today if I take part in this study?**

We would like to ask you to take part in a interview. We are interested to hear about your experiences and opinions; there are no right or wrong answers. This will take around 60 minutes.

**What will happen to my data?**

The information that you share with us is confidential and will be kept securely with limited access. No one will be able to identify you based on what you share with us. However, de-identified data collected from this study may also be shared with other groups of researchers.

**What are the benefits to taking part in this interview?**

There are no personal benefits to taking part in this interview, but the benefit of this study is to improve appropriateness of antimicrobial prescribing to the patients and to increase the local quality improvement expertise and be able to subsequently improve other areas of the care.

**What risks can I expect from taking part in the interview?**

Participation in any research study may involve some loss of privacy, and you may feel uncomfortable answering some questions. We will fill your answers into a questionnaire, but your name will not be used in any reports, and your information will be anonymized. Should you feel uncomfortable with any question, you are free to skip it or to withdraw your statements at any time without negative consequences for you.

**Do I have to take part in this interview?**

No, it is your choice – your participation in this study is entirely voluntary. If you decline, this will not impact your future career. You can also choose not to answer some questions if you feel uncomfortable, or you can stop giving answers altogether at any time – it is your choice to refuse or to participate. If you wish to withdraw, the researcher will dispose of your data accordingly.

**Who can answer my questions about the study?**

If you have questions, complaints, or concerns about the research process or if any undesirable risk occur to you, you can contact …...*(local EC contact details)*….
