# Supplementary material for: Evidence based QUality Improvement for Prescribing Stewardship in ICU (EQUIPS-ICU): protocol for type III hybrid implementation-effectiveness study
Source: Implement Sci. 2025 Feb 25;20:12. doi: 10.1186/s13012-024-01413-4 (PMC11863957; doi:10.1186/s13012-024-01413-4)
Supplement: Supplementary file 6 — Supplementary Material 6. Record of oral consent. [file 13012_2024_1413_MOESM6_ESM.docx]

**RESEARCHER RECORD OF ORAL CONSENT**

**Study Title:** Evidence based QUality Improvement for Prescribing Stewardship in ICU

(EQUIPS- ICU). Can a structured antimicrobial review be implemented in

LMIC ICUs?

**Short Title:** Implementing a structured antimicrobial review in LMIC ICUs

**Investigators:** Dr Duncan Wagstaff, Dr Abi Beane

**Participant Name: __________________________**

**Date (DD/MM/YYYY):** ___________________________

**Has the project been explained to the Participant?**

□ Yes □ No

**Has the Participant received the Participant Information Sheet?**

□ Yes □ No

**Has the Participant agreed to be interviewed?**

□ Yes □ No

**Has the Participant agreed to data to be shared?**□ Yes □ No

**Has the consent process been audio recorded?**

□ Yes □ No

**Name of Researcher:** ______________________________

**Signature of Researcher:**

(Signed in the presence of the Participant to confirm oral consent
