# Supplementary material for: Evidence based QUality Improvement for Prescribing Stewardship in ICU (EQUIPS-ICU): protocol for type III hybrid implementation-effectiveness study
Source: Implement Sci. 2025 Feb 25;20:12. doi: 10.1186/s13012-024-01413-4 (PMC11863957; doi:10.1186/s13012-024-01413-4)
Supplement: Supplementary file 8 — Supplementary Material 8. List of redundant combinations of antimicrobials. [file 13012_2024_1413_MOESM8_ESM.docx]

**Redundant combinations of antimicrobials**

The concurrent administration of two or more of any of the following drugs within the three groups (i.e. two drugs within Gram negative group etc)

| Groups | Drugs |
| --- | --- |
| Gram negative | cephalosporins (cefuroxime, ceftriaxone, ceftazidime, cefotaxime, and cefepime) |
|  | fluoroquinolones (ciprofloxacin and levofloxacin) |
|  | β-lactam plus β-lactamase-inhibitor combinations (amoxicillin/clavulanate and piperacillin/tazobactam) |
|  | aminoglycosides (amikacin, gentamycin, and tobramycin) |
|  | carbapenems (meropenem, ertapenem, doripenem, and imipenem) |
|  | tigecycline |
|  |  |
| Gram positive | β-lactams (amoxicillin, amoxicillin-clavulanate, cefazolin, and cloxacillin) |
|  | tigecycline |
|  | clindamycin |
|  | linezolid |
|  | glycopeptides (vancomycin and teicoplanin) |
|  |  |
| Anti-anaerobic | metronidazole |
|  | β-lactam plus β-lactamase-inhibitor combinations (amoxicillin/clavulanate and piperacillin/tazobactam) |
|  | carbapenems (meropenem, ertapenem, doripenem, and imipenem) |
|  | moxifloxacin |
|  | clindamycin |
|  | cefoxitin |
|  | tigecycline |
