# Supplementary material for: Evidence based QUality Improvement for Prescribing Stewardship in ICU (EQUIPS-ICU): protocol for type III hybrid implementation-effectiveness study
Source: Implement Sci. 2025 Feb 25;20:12. doi: 10.1186/s13012-024-01413-4 (PMC11863957; doi:10.1186/s13012-024-01413-4)
Supplement: Supplementary file 7 — Supplementary Material 7. OxTrec minimal risk approval letter. [file 13012_2024_1413_MOESM7_ESM.pdf]

Oxford Tropical Research Ethics Committee

University of Oxford
Research Services, Research Governance, Ethics & Assurance
Boundary Brook House, Churchill Drive, Oxford OX3 7GB
Tel. +44 (0)1865 (2)82106
E-mail: oxtrec@admin.ox.ac.uk

11 September 2024

Dr. Duncan Wagstaff
Mahidol Oxford Tropical Medicine Research Unit (MORU)
Bangkok
Thailand

Dear Dr. Wagstaff,

Full Title of Study: Evidence based QUality Improvement for Prescribing Stewardship in ICU (EQUIPS-ICU). Can a structured antimicrobial review be implemented in LMIC ICUs?

OxTREC Reference: 559-24

Thank you for your email of 23 August 2024 and updated minimal risk study application and documents.

I am pleased to confirm that approval has now been granted for this study. This is valid for the planned duration of the study as detailed in the application and is subject to receiving the local ethical approval (if this approval has not yet been received).

The documents approved for this study are as follows:

**Documents:**	**Version:**	**Date:**
Minimal risk application form	V2.0	20/08/24
PIS	V2.0	20/08/24
Record of oral consent form	V2.0	20/08/24
Appendix 1: Baseline Assessment	V2.0	20/08/24
Appendix 2: Interview guide month 4-6	V2.0	20/08/24
Appendix 3: Interview guide month 7	V2.0	20/08/24

Any subsequent changes to the application must be submitted to the Committee as an Amendment. This should include a letter to give the reasons for the proposed modifications and all revised documents with changes tracked.

Please ensure that you submit a completed Annual Report form on every anniversary of this approval and a final End of Study Report. The relevant forms can be found on the OxTREC website.

Finally, please note the following **important information**:

Data safety—all studies
It is the responsibility of the PI to ensure that all data collected during the course of the study is stored and transferred safely and securely. Further guidance and advice are available from the Research Data Team.

Only studies that will involve storing human tissue samples in Oxford
If you are planning to import the samples into England, you will need to make arrangements before

the samples are transferred to store them under the governance of a Human Tissue Authority (HTA) licence. It is a legal requirement that any tissue or fluid made up of or containing human cells to be used for the purpose of research is stored on premises licensed by the HTA unless covered by an exemption. OxTREC approval is not a recognised exemption. Further information may be found on the University's human tissue governance web pages.

Yours sincerely,

DocuSigned by:

Sami Kelsh

B4D7A3EB6876444...

Sami Kelsh
Research Ethics Administrator, OxTREC

for
Dr. Rosemary Musesengwa
Research Ethics Manager, OxTREC
